# A model for greed cells in 3-D environments

**DOI:** 10.1186/1471-2202-14-S1-P396

**Published:** 2013-07-08

**Authors:** Federico Stella, Bailu Si, Emilio Kropf, Alessandro Treves

**Affiliations:** 1 Cognitive Neuroscience Sector, Sissa, Trieste, Italy; 2 Department of Neurobiology, Weizmann Institute of Science, Rehovot, Israel; 3 Laboratory of Neuronal Plasticity, Leloir Institute, Buenos Aires, Argentina; 4 Embassy of Italy, Science Office, Tel Aviv, Israel

## 

Individual medial entorhinal cortex (mEC) 'grid' cells provide a representation of space that appears to be essentially invariant across environments, modulo simple transformations, in contrast to multiple, rapidly acquired hippocampal maps; it may therefore be established gradually, during rodent development. lf this is the case, then the topology of the environment in which the development takes place should affect the way the grid final configuration appears.

Until now, models of grid cells have dealt only with planar, two-dimensional topologies.

We extend our single-cell adaptation model [1] to include the third dimension in the environment. We study two non-planar topologies:

- the sphere surface [2]

- the fully three-dimensional space.

What grid cell firing maps would we expect to observe?

In the first condition the model predicts a sequence of spherical harmonics, each of which is the optimal asymptotic solution for the self-organizing adaptation process, within a certain range of the world radius. In the second, it predicts that the grid fields should assume a configuration analogous to an hexagonal close-packing lattice.

New experiments that make use of virtual reality on a revolving sphere, or in which rats are reared inside a spherical cage, will likely be a direct test for our model on the sphere, while experiments with flying bats will provide evidence on the feasibility of a genuine 3-dimensional representation of space in terms of grid units.

## References

[B1] Emilio Kropff Alessandro Treves The emergence of grid cells: intelligent design or just adaptation? Hippocampus 2008 19021261 10.1002/hipo.20520

[B2] Federico Stella Bailu Si Emilio Kropff Alessandro Treves Grids cells on the ball JSTAT 2012

